# Reconciliation or reputation: critical analysis of commercial sector commitments and framing in reconciliation action plans

**DOI:** 10.1093/heapro/daag061

**Published:** 2026-05-08

**Authors:** Petrina Leersen, Bronwyn Fredericks, Andrew D Brown, Jennifer Browne, Yin Paradies

**Affiliations:** Faculty of Health, Deakin University, Institute for Health Transformation, Global Centre for Preventive Health and Nutrition (GLOBE), 1 Gheringhap Street, Geelong, VIC 3220, Australia; Office of the Deputy Vice-Chancellor (Indigenous Engagement), The University of Queensland, Brisbane, QLD 4072, Australia; ARC Centre of Excellence for Indigenous Futures, UQ Poche Centre for Indigenous Health; Faculty of Health, Deakin University, Institute for Health Transformation, Global Centre for Preventive Health and Nutrition (GLOBE), 1 Gheringhap Street, Geelong, VIC 3220, Australia; Faculty of Health, Deakin University, Institute for Health Transformation, Global Centre for Preventive Health and Nutrition (GLOBE), 1 Gheringhap Street, Geelong, VIC 3220, Australia; Faculty of Arts and Education, Deakin University, Institute for Citizenship and Globalisation, 1 Gheringhap Street, Geelong, VIC 3220, Australia

**Keywords:** reconciliation action plan, commercial determinants of health, First Nations, Aboriginal and Torres Strait Islander health, corporate social responsibility, frame analysis, health equity

## Abstract

Reconciliation action plans (RAPs) are increasingly used by commercial organizations in Australia to demonstrate commitment to Aboriginal and Torres Strait Islander peoples. Framed as tools to promote understanding and address historical injustice, these documents also serve organizational interests, particularly in reputation management and corporate social responsibility (CSR). This research critically examined the structure, practices, portfolios, resources, and transparency of high-level RAPs of for-profit organizations operating in the state of Queensland, Australia. Drawing on frame analysis, critical discourse analysis (CDA), and commercial determinants of health frameworks, this study explored how reconciliation and associated commitments are defined, justified, and operationalized within corporate discourse. The analysis showed that resource-rich corporations leveraged their power to manage reconciliation as a corporate asset rather than address structural inequities. Five overlapping frames, ‘instrumental reconciliation, performative accountability, symbolic leadership, truth signalling, and substantive recognition’, were identified through which reconciliation is positioned within corporate discourse of legitimacy, measurement, and control. While many organizations adopt languages of codesign, self-determination, and cultural safety, translation into demonstrable processes and measurable outcomes remains inconsistent and limited. These findings highlight how RAPs can both provide entry points for engagement while simultaneously reinforcing existing power relations. The research contributes to understanding the commercial determinants influencing Aboriginal and Torres Strait Islander health and wellbeing, calling for stronger accountability, transparency, and Aboriginal and Torres Strait Islander leadership within corporate reconciliation agendas.

Contribution to Health Promotion

- Reconciliation action plans (RAPs) align with broader environmental, social, and governance (ESG) and CSR strategies that organizations use to signal commitments to Aboriginal and Torres Strait Islander health.
- From a commercial determinants of health perspective, RAPs often prioritize corporate reputation over structural reconciliation change.
- Our analysis reveals gaps between symbolic commitments and substantive action.
- Reconciliation action plan reporting practices limit transparency and accountability.

## Introduction

Aboriginal and Torres Strait Islander people are the Indigenous peoples of Australia (United Nations General Assembly 2007), with distinct cultural, linguistic, and social systems that have existed for tens of thousands of years. European colonization in Australia was characterized by dispossession, genocide, violence, forced child removals, and assimilation policies, causing profound and ongoing social, economic, and health inequities (Quayle and Sonn 2019, Paradies 2020, Sharpe *et al*. 2022). Colonial structures imposed Western political, economic, and legal systems, underpinned by racist ideologies and cultural imperialism, which continue to devalue Indigenous knowledges, disrupt traditional practices, and restrict access to land and resources (Tomlinson 2012, Williams Lawrence and Davis 2019). These historical and ongoing processes contribute to disparities in life expectancy, chronic disease prevalence, mental health outcomes, and socioeconomic status between Aboriginal and Torres Strait Islander peoples and non-Indigenous Australians (Griffiths *et al*. 2016, Australian Institute of Health and Welfare 2024, Australian Institute of Health and Welfare 2025, Dudgeon *et al*. 2025).

In the Australian context, colonialism and the rise of free-market capitalism have entrenched structural conditions that produce inequities. For example, a survey of over 1000 Aboriginal and Torres Strait Islander workers found that 38% experienced unfair treatment, 44% heard racial slurs, 59% experienced racism about their appearance, and 63% felt pressured in expressing their identity (Diversity Council Australia 2020). Racism is recognized as a determinant of health, contributing directly and indirectly to adverse physical and mental health outcomes (Paradies *et al*. 2015).

These inequities are further shaped by the commercial determinants of health (CDoH), defined as the ‘systems, practices, and pathways through which commercial actors drive health and equity’ (Gilmore *et al*. 2023). Corporations can both contribute to health harms and offer opportunities for economic empowerment, but power imbalances, systemic racism, and inadequate regulation often amplify risks for Aboriginal and Torres Strait Islander communities (Anaf *et al*. 2020, Sharpe *et al*. 2022). Lacy-Nichols and colleagues (2023) proposed a framework for examining commercial entities’ impact on public health through their practices, portfolios, resources, organizational structure, and transparency to allow further examination of whether, in what ways, and to what extent a commercial actor may influence health outcomes.

In recent years, several high-profile cases have highlighted how commercial practices disproportionately harm Aboriginal and Torres Strait Islander peoples. These include Rio Tinto’s destruction of sacred rock caves at Juukan Gorge (2020) (Holcombe and Fredericks 2021, Oliveri *et al*. 2024), Telstra’s AUD 50 million penalty for selling unsuitable contracts to Aboriginal and Torres Strait Islander people (2021) (Australian Competition and Consumer Commission 2022), and Woolworths’ attempt to open a Dan Murphy’s alcohol superstore near alcohol-free communities after a 5-year regulatory battle (2021) (Crocetti *et al*. 2023a). These examples, among others, triggered a parliamentary inquiry into corporate sector engagement with Aboriginal and Torres Strait Islander peoples (Leersen *et al*. 2024). Submissions to this inquiry revealed numerous systems, practices, and pathways through which the commercial sector may harm Aboriginal and Torres Strait Islander health, including the supply of harmful products, misleading branding, and exploitative consent processes, resulting in financial stress, poor mental health, and restricted access to essential services (Leersen *et al*. 2024).

The nascent research on the commercial determinants of Aboriginal and Torres Strait Islander health includes a synthesis of international Indigenous health literature (Crocetti *et al*. 2022), analysis of Australian media articles (Crocetti *et al*. 2023b), interviews with Aboriginal leaders from Victoria (Crocetti *et al*. 2024), analysis of submissions to the above-mentioned inquiry (Leersen *et al*. 2024), and a case study of the proposed Dan Murphy’s alcohol store in Darwin (Crocetti *et al*. 2023a). However, there is limited research on the CDoH in Queensland, where experiences may differ from other regions. Queensland encompasses both mainland regions and the Torres Strait Islands, with a high proportion of rural and remote communities. According to the 2021 Census, 237 000 people identified as Aboriginal and Torres Strait Islander, representing 4.6% of the state’s population and the second largest Aboriginal and Torres Strait Islander population nationally (Australian Bureau of Statistics 2022). Queensland also has a diverse commercial landscape of mining, tourism, retail, and telecommunications, which makes it a critical site to examine how commercial practices, allocation of resources, and transparency influence Aboriginal and Torres Strait Islander health equity.

Australia’s formal reconciliation agenda began in 1991 with the establishment of the Council for Aboriginal Reconciliation (Fredericks Smith and Mann 2017). A decade later, Reconciliation Australia was founded as an independent not-for-profit organization, and in 2006, it introduced the Reconciliation Action Program (Schepis 2020, Reconciliation Australia 2024). Reconciliation Australia defines Reconciliation as ‘about strengthening relationships between Aboriginal and Torres Strait Islander peoples and non-Indigenous peoples, for the benefit of all Australians’ (Reconciliation Australia 2025b). Reconciliation action plans (RAPs) were introduced as a structured way for organizations to translate ‘good intentions into action’ contributing to the national reconciliation movement (Reconciliation Australia 2023). The programme includes four progressive RAP types: Reflect, Innovate, Stretch, and Elevate (Reconciliation Australia 2023). High-level RAPs, Stretch and Elevate, are designated for organizations positioned as national leaders, with expectations to promote Aboriginal and Torres Strait Islander self-determination and drive transformational, systemic change (Reconciliation Australia 2023). While RAPs are specific to the Australian reconciliation context, they resemble other corporate social responsibility (CSR) initiatives through voluntary participation, organizational self-definition, and public signalling of social responsibility.

RAPs have since been adopted by hundreds of organizations, including large commercial entities operating in industries, such as mining, retail, banking, and telecommunications, which hold significant social, economic, and political influence. Promoted as a framework for building respect, trust, and opportunity, RAPs aim to embed culturally responsive actions into organizational structures and operations. As the programme has expanded, however, concerns have emerged about the sincerity and substance of these commitments. RAPs are not neutral. They can function as instruments of corporate reputation management, enabling companies to bolster their public image while avoiding deeper structural change (Crocetti *et al*. 2024).

Previous research examining RAPs has found mixed perspectives among Aboriginal and Torres Strait Islander peoples. While some studies reported that RAPs were viewed as genuine attempts at relationship building and accountability, others have critiqued them as tokenistic, reinforcing corporate colonialism, and displacing responsibility for reconciliation onto Indigenous employees (Crocetti *et al*. 2024, Leersen *et al*. 2024). Additional critiques note that RAPs rarely advance Indigenous sovereignty or self-determination (Coalition of Aboriginal and Torres Strait Islander Peak Organisations and Australian Governments 2020; Weerasinghe Chapple and Williamson 2023). Despite more than 3000 organizations holding RAPs, it remains unclear whether they deliver substantive benefits or primarily serve commercial interests (Reconciliation Australia 2025a).

This study aims to critically examine the RAP commitments made by Australian commercial entities operating in Queensland through a commercial determinant of health lens. Specifically, it seeks to answer two research questions:

1. To what extent do RAP commitments align with organizations’ structures, practices, portfolios, resources, and transparency and what are the implications for Aboriginal and Torres Strait Islander health and equity?
2. How do Queensland-based commercial organizations with high-level RAPs frame their reconciliation commitments?

By analysing how commercial actors define problems, assign responsibility, make moral claims, and propose solutions, this study deepens understanding of the private sector’s role in promoting or undermining Aboriginal and Torres Strait Islander health equity. Although it does not assess health outcomes directly, it offers insight into the mechanisms through which corporations influence reconciliation and the commercial determinants of Aboriginal and Torres Strait Islander health.

## Materials and methods

### Theoretical framework

This study was informed by a constructionist epistemology, which views social reality as coproduced through cultural, historical, and interpersonal processes (Vera 2016), and guided by critical social theory, which foregrounds power, inequality, and the social construction of knowledge (Finlayson and Huw Rees 2023; McCall *et al*. 2023). The research team comprised social workers, public health researchers, and senior Aboriginal academics, bringing together diverse disciplinary and lived perspectives grounded in a shared commitment to examining the commercial determinants of Aboriginal and Torres Strait Islander health.

The analysis was guided by Entman’s (1993) framing theory, which examines how aspects of reality are selected to define problems, attribute causes, make moral evaluations, and propose remedies. In this study, it was used to analyse how reconciliation was conceptualized across RAPs, including how problems, responsibility, values, and solutions were framed. A critical discourse analysis (CDA) lens was also applied to examine how language functions within RAPs, not only to describe commitments but also to perform social, political, and ideological work. CDA highlights what is said, how it is said, and what is left unsaid, foregrounding power relations and the strategic use of language to manage reputation and construct legitimacy (Leotti Sugrue and Winges-Yanez 2022). Combining framing with CDA enabled examination of not just what RAPs say, but how they say it, for what purpose, and with what effects.

### Data collection

**Figure 1:**
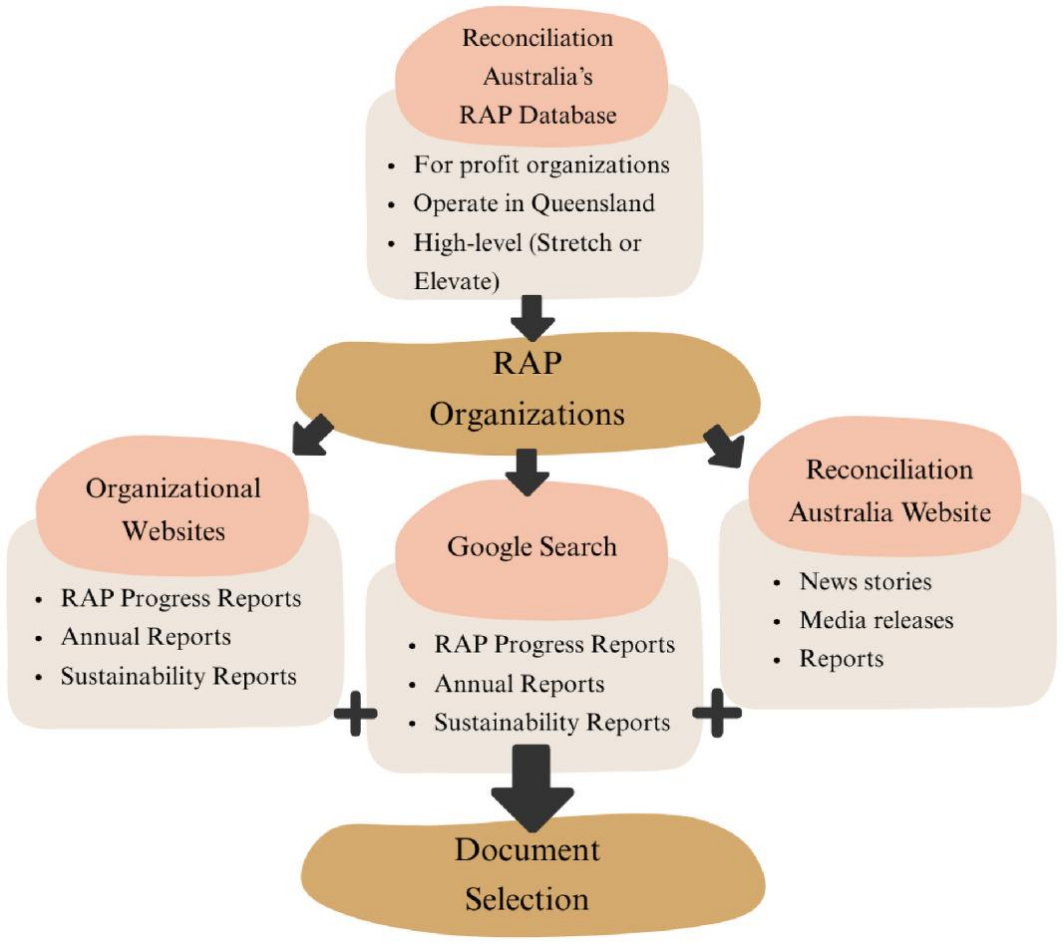
Process for data collection.

This study focused on private sector organizations operating in Queensland with Stretch or Elevate RAPs, the two highest RAP levels. Stretch and Elevate RAPs are designed for organizations positioned as leaders in reconciliation and are characterized by higher expectations for accountability, transparency, and the implementation of actions intended to drive systemic change and support Aboriginal and Torres Strait Islander self-determination (Reconciliation Australia 2023). Data sources included three types of documents (Fig. 1).

First, Reconciliation Australia’s website was searched using filters for RAP type, state, and sector to identify RAP organizations that met the inclusion criteria:

1. Have a Stretch or Elevate level RAP.
2. Private sector organization.
3. Operate in Queensland, including both Queensland-based organizations and national organizations operating in Queensland.

For each selected organization, its RAP was downloaded from the Reconciliation Australia website. Organizations’ websites were then reviewed for details on RAP implementation by searching for key terms including ‘reconciliation’, ‘Aboriginal’, ‘Torres Strait Islander’, ‘Indigenous’, ‘First Peoples’, ‘First Nations’, and ‘RAP progress report’. This produced a series of documents, including RAP progress reports, annual reports, and sustainability reports. Website searches were complemented by an additional Google search using the format ‘Organization Name + RAP progress report’. Where multiple reports were available, only the most recent was downloaded for analysis.

Finally, Reconciliation Australia’s website was searched for current and historical news stories, media releases, reports, and other publications relating to RAP organizations, highlighting both achievements and setbacks, such as RAP revocations. These documents provided context on how RAPs operate and revealed public perceptions that may influence how companies frame themselves within their RAPs. Data collection was completed in May 2025.

### Data analysis

Selected documents were imported into NVivo qualitative analysis software for coding. Documents that were only available online and could not be uploaded to NVivo were coded directly in Microsoft Excel. Coding was conducted in two phases, guided by the research questions. In the first phase, the Commercial Entities and Public Health Framework (Lacy-Nichols *et al*. 2023) was used to code details about organizations’ portfolios, resources, and transparency. This phase of analysis focused on identifying what commitments organizations made and how these aligned with key CDoH domains.

In the second phase, documents were inductively coded, drawing on framing theory and CDA. Framing theory guided attention to how organizations defined problems, attributed responsibility, articulated moral claims, and proposed solutions within their RAPs and related documents. CDA focused on the language, narratives, and discursive strategies used to construct reconciliation, including the use of legitimizing language, omissions, passive constructions, and representations of power. Initial coding and frame construction were undertaken by the first author, with initial findings reviewed and discussed with coauthors as part of an iterative analysis process.

### Ethical approval

This study received an ethics exemption from the Deakin University Human Research Ethics Committee (2024/HE000861) as all data were publicly available.

## Results

### Sample characteristics

**Figure 2:**
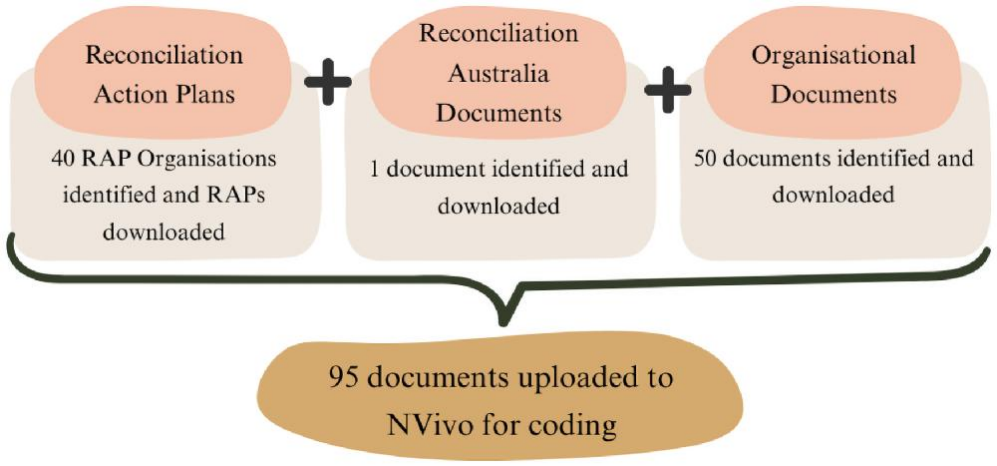
Document selection.

Forty RAP organizations and 84 individual documents were included in the analysis (Fig. 2). With consideration for the time-sensitive nature of RAPs, all documents included in the analysis reflect the most recent search conducted on 8 May 2025. Documents included 33 Stretch and seven Elevate level RAPs, five RAP progress reports, 50 other organizational documents including annual reports or ESG reports, and one document from Reconciliation Australia. In the sections below, RAP organizations are referred to in parentheses using de-identified codes, e.g. Org1 and Org2.

### Organizational structure

RAP organizations included 23 privately owned and 17 publicly listed companies. Geographic reach varied: 21 organizations operated solely in Australia, three were Australasian, and 16 were multinational, including subsidiaries in multiple regions (see Supplementary File 1). Despite the scale of many of these organizations, Aboriginal and Torres Strait Islander representation at executive levels was almost entirely absent (see Table 1). Only one organization reported a First Nations executive, which was also the smallest organization in the dataset by both employee numbers and reported annual revenue.

**Table 1:** Aboriginal and Torres Strait Islander leadership representation and RAP governance structures.

Aboriginal and Torres Strait Islander leadership representation and RAP governance structures	No. of organizations
Aboriginal and/or Torres Strait Islander Board/Executive Members	1
RAP Working Group/RWG/Reconciliation Working Group	22
RAP Committee/RAP Steering Committee	10
Indigenous Advisory Group/Advisory Board/Advisory Council	6
RAP Leadership Team/Leadership Circle	3
Expert Panel/Reference Group/Indigenous governance body	2
Board/Executive oversight structures linked to RAP governance	8
Not listed/unclear RAP structure	1

All organizations reported internal governance structures related to their RAPs. These included RAP working groups or committees to oversee RAP implementation and progress. These groups typically coordinated cultural awareness activities, special events (e.g. NAIDOC week), and community engagement initiatives. Limited information was available regarding the decision-making power or governance of these working groups. One company (Org6) mentioned independent advisory groups, but this tended to focus on development stages of the RAP rather than monitoring and accountability.

### Portfolio and practices

**Figure 3:**
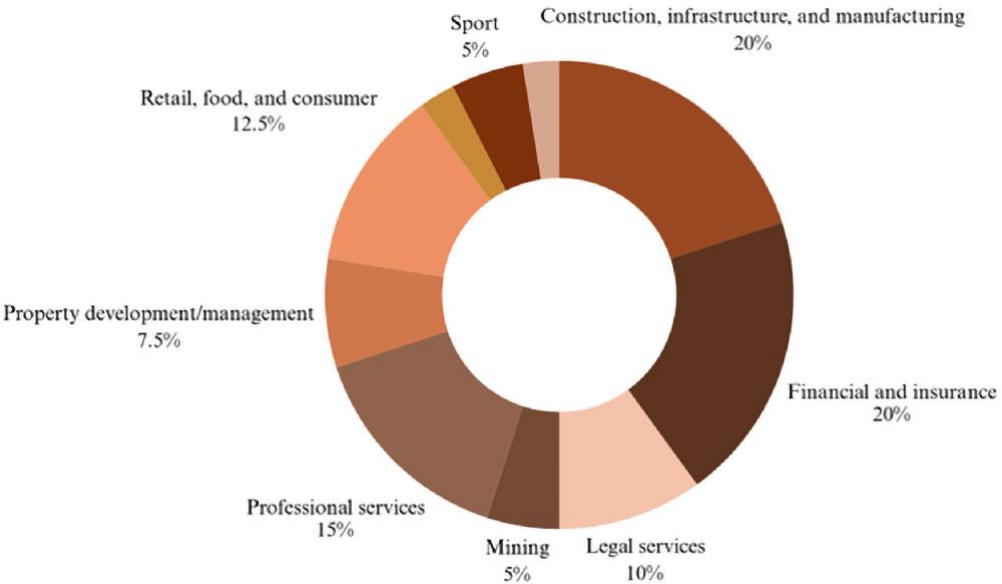
Industry sector of RAP organizations.

The classifications used in this analysis followed Reconciliation Australia’s industry categories. Most organizations with high-level RAPs were from the construction, infrastructure, and manufacturing industries, as well as the finance and insurance industries (*n* = 8 each), followed by professional services (*n* = 6) and retail, food, and consumer industries (*n* = 5) (Fig. 3). Finance and insurance companies included major banks, superannuation funds, and financial and retirement advisory firms, some linked to healthcare and community services. Retail and consumer companies provided catering, cleaning, and facility management, as well as grocery and alcohol retail. Professional services covered auditing, consulting and employment firms operating across justice, immigration, health, and defence.

While some sectors with significant influence over social and commercial determinants of Aboriginal and Torres Strait Islander health, including tobacco, alcohol, and ultraprocessed food, were not identified in this work, retailers selling these products were included, alongside companies involved in mining and prisons.

The legal firms identified (Org18–21) are included in the top ranked for Native Title proponents, the practice of representing companies, government bodies, or individuals seeking to carry out projects, such as mining or infrastructure on land, where Native Title rights exist (Chambers and Partners 2025). However, in their RAPs, the firms only highlighted pro bono practice for First Nations clients in their commercial portfolios.

### Resources

Organizational size and financial resources varied considerably. Employee numbers ranged from 700 (Org24) to 125 208 (Org28), with one organization as an outlier, employing only 10 staff (Org40). Reported annual revenues ranged from AUD 43.7 million (Org38) to AUD 43.66 billion (Org34). Specific details regarding resourcing of reconciliation commitments were limited. Funding was most often dedicated to employment or procurement strategies rather than systemic reconciliation initiatives.

### Transparency

All organizations maintained publicly accessible RAP documents on their websites and through Reconciliation Australia’s website. However, the accessibility of these documents varied considerably. RAPs were often difficult to locate without using website search functions; 14 required navigating through at least five website layers with no consistent placement or direct links. The RAP documents themselves varied widely in length, ranging from 15 pages (Org6) to 80 pages (Org35).

Most organizations incorporated RAP progress within broader sustainability or annual reports. Five organizations provided dedicated RAP progress reports (Org7, 15, 21, 27, 26). Overall, RAP progress reports provided the most detailed measures of progress and offered the greatest potential for monitoring and accountability through transparency; however, the quality and depth of reporting varied. For example, some progress reports included only the number of commitments progressed or achieved (Org21), two provided more detailed progress updates on how commitments were achieved (Org15, 36), and two reported updates on every commitment, including those not achieved (Org7, 27). Some multinational organizations included Indigenous populations outside Australia in their RAP reporting (e.g. New Zealand and Canada) and occasionally reported international Indigenous employment figures; however, comparable data for Australia were often absent.

Several organizations reported Aboriginal and Torres Strait Islander employment figures in ways that made assessment difficult or potentially misleading. For example, one organization set a 3.2% employment target by 2026 but publicly reported 1.2% in both 2023 and 2024, while its 2024 RAP Progress Report stated that the target was ‘achieved’ (Org15). Another celebrated: ‘whilst this representation is below the external AU benchmark of 3.2%, this has doubled from 0.4% in 2019’ (Org12). Some organizations provided only absolute numbers rather than percentages, such as committing to employing ‘1200 more young Aboriginal and Torres Strait Islander people over three years’ within a workforce of ∼120 000 people (Org35), limiting interpretability and comparison over time. No organizations provided breakdowns of their reported employment numbers by employment type (full-time, part-time, casual, permanent, temporary) or classification (trainees, apprentices, graduates, technical officers, supervisors).

### Framing overview

Five overlapping frames were constructed from the data: ‘instrumental reconciliation, performative accountability, symbolic leadership, truth signalling, and substantive recognition’. The major frames, associated subframes, and framing functions are shown in Table 2, along with illustrative quotes from RAPs.

**Table 2:** Frame overview.

Frame 1	Instrumental reconciliation		
Framing functions	Organizational initiative for reputation enhancement		
Subframes	‘Symbolic inclusion’	‘Aesthetic reconciliation’	‘Business-as-usual reconciliation’
Framing functions	Any collaboration framed as proof of inclusion	Visual symbolism used to signal cultural respect	Aligned with existing corporate agendas
**Frame 2**	**Performative accountability**		
Framing functions	The process of reporting is presented as accountability		
Subframes	‘Sanitized history’	‘Cultural endurance’	‘Displaced responsibility’
Framing functions	Vague historical acknowledgements framed as accountability	Aboriginal and Torres Strait Islander resilience, continuity, and survival as success	Commitments framed as contingent or flexible
**Frame 3**	**Symbolic leadership**		
Framing functions	Organizations position themselves as leaders		
Subframes	‘External/community-focused leadership’	‘Pathway leadership’	‘Ambiguous leadership’
Framing functions	Engaging external actors (e.g. consultants) over internal, sustained RAP leadership	Leadership framed as achieved through long-term pathways (e.g. internships), rather than direct employment at senior levels	Leadership is a flexible term
**Frame 4**	**Truth signalling**		
Framing functions	Support truth telling in a depoliticized and nonconfrontational manner		
**Frame 5**	**Substantive recognition**		
Framing functions	Acknowledges specific organizational harms and negative impacts		

### Instrumental reconciliation

The ‘instrumental reconciliation’ frame constructed reconciliation and RAPs as mechanisms for securing and maintaining organizational legitimacy. Within this frame, reconciliation aligned company strategy with public values and stakeholder expectations, enhancing brand identity and corporate image. Rather than emphasizing social justice or power sharing, RAPs fulfilled risk management and human resource objectives, signalling cultural awareness and goodwill without challenging internal power structures. RAP commitments were rich in symbolic power, expressed in managerial language but conservative in practice, positioning RAPs to serve organizational objectives and operations rather than advancing broader structural goals of reconciliation. For example, organizations described reconciliation through managerial initiatives, such as ‘integrating First Nations Affairs into the ESG function’ (Org9) and stating they ‘report on the progress we have made as good corporate citizens’ (Org28), framing reconciliation as something delivered through existing corporate structures rather than structural change.

While reconciliation was often framed as a long-term, reflective, and participatory process, there were examples within the documents of reconciliation framed as a destination, a point where a ‘reconciled Australia’ will be achieved (Org7, 15, 29, 35, 37). This goal orientation centred tangible actions, such as cultural safety training, employment opportunities and targets, and procurement as evidence of progress, suggesting organizations viewed their role in reconciliation as a checklist of commitments rather than part of broader transformative change.

Many organizations used reconciliation as part of their branding, linking RAPs and reconciliation to their values, ethics, and public image using language, such as ‘doing the right thing’ (Org7, 12) or being a ‘[good] corporate citizen’ (Org3, 18, 27). Others integrated it directly into business strategy, e.g. ‘[reconciliation] is central to our Win Together strategy’ (Org34) or ‘business is only as successful as the community in which it operates’ (Org21). These examples reflect instrumental reconciliation, where social responsibility was framed as good for business, building social credibility while protecting commercial interests. Within this frame, there were three subframes: ‘symbolic inclusion, aesthetic reconciliation, and business-as-usual reconciliation’.

#### Symbolic inclusion

The ‘symbolic inclusion’ frame extends from ‘instrumental reconciliation’ by demonstrating how organizations used popular progressive language, such as codesign or inclusivity, to appear culturally responsive and forward thinking without practising what those words imply. The discourse of codesign featured prominently, with many RAPs presenting codesign as the pinnacle of collaboration and respect (Org1, 6–7, 12–13, 17, 23, 27, 29–30). One RAP used the term ‘codesign’ 17 times (Org33). However, in most cases, the term was applied to activities that resembled consultation or joint delivery rather than genuine cogovernance or shared decision-making. Here, codesign was framed as a method for improving cultural appropriateness, not as an approach that redistributes decision-making power. References such as ‘codesign areas which can be improved’ (Org23) or ‘codesign sustainable initiatives’ (Org12) were vague and provided little detail about how, or if, power was shared. Within the frame of ‘instrumental reconciliation’, this language symbolically included Aboriginal and Torres Strait Islander voices while ensuring reconciliation remained organizationally managed.

Similarly, the discourse of intersectionality was deployed in a handful of RAPs. Intersectionality was often used to demonstrate inclusivity without linking it to the organization’s own role in contributing to or addressing intersectional inequities. For example, one progress report acknowledged that ‘First Nations LGBTIQA + SB people are exposed to compounding racist, homophobic and/or transphobic discrimination’ (Org27).

#### Aesthetic reconciliation

A feature across all RAP documents was the prominent use of Aboriginal and Torres Strait Islander artwork. This visual emphasis, termed ‘aesthetic reconciliation’, functioned as a way for organizations to visibly demonstrate inclusion (Org1–40). However, this cultural symbolism often functioned as a performative strategy, allowing organizations to appear committed to reconciliation while avoiding deeper engagement with issues of power, voice, shared decision-making, Indigenous self-determination, and systemic change. At times, the artwork appeared to be co-opted by organizations, with corporate branding and language incorporated into its meaning (Org1–5, 10–11, 15, 17, 23, 25, 32, 38–39). These organizations referred to Aboriginal and Torres Strait Islander artistic designs through the lens of their organization describing cultural symbols using corporate terms. Examples included ‘these boomerangs represent [organization’s] core business’ (Org32), ‘design visually expresses our refreshed brand and values’, and ‘the meeting places with paths reaching out depict [organization’s] partners and clients’ (Org3). This corporate framing distances the art from the artist’s lived experience and Aboriginal and Torres Strait Islander cultural context. Additionally, one organization credited the artwork to a business without acknowledging individual artists and used Aboriginal and Torres Strait Islander designs without explanation (Org7). This raised questions about who benefited from the use of the artwork and whether it genuinely supported Aboriginal and Torres Strait Islander peoples or simply serviced organizational branding.

#### Business-as-usual reconciliation

Within ‘business-as-usual reconciliation’, organizations advanced reconciliation by highlighting activities they were already legally or operationally required to do, presenting compliance and harm mitigation measures as reconciliation commitments. Mandatory or risk management activities were reframed as reconciliation achievements, including in areas where their industries are implicated in harm. Extractive and infrastructure companies presented statutory cultural heritage processes as contributions to reconciliation, positioning themselves as protectors of heritage even while these laws constrain their operations. Routine regulatory engagement was recast as relationship building, e.g. describing land clearing works as ‘working alongside Traditional Owners’ (Org22), or compliance processes as ‘supporting the preservation of cultural heritage’ (Org23). Financial institutions similarly elevated standard commercial practices as reconciliation outcomes, positioning self-determination as wealth generation delivered through their own products (Org15). Across sectors, reconciliation was used to legitimize existing business practices.

Employment was frequently positioned as a primary expression of reconciliation (Org1, 14, 25, 35). Employment targets, traineeships, and recruitment initiatives were presented not only as practice actions but also as evidence of organizational commitment, showcasing reconciliation in ways easily quantifiable and reputationally advantageous. In this framing, reconciliation was achieved through workforce inclusion.

Some organizations recognized that barriers, such as having a criminal record, could disproportionately affect Aboriginal and Torres Strait Islander people and adjusted hiring policies accordingly, acknowledging that commercial practices can reinforce inequities, e.g. through hiring policies and compliance checks that disproportionately exclude Aboriginal and Torres Strait Islander applicants (Org1, 29). Others simply aimed to be an ‘attractive place to work for First Nations [staff]’ without diagnosing the structural barriers contributing to underrepresentation of Aboriginal and Torres Strait Islander peoples in their workforce (Org24).

### Performative accountability

The ‘performative accountability’ frame utilized RAPs to acknowledge harms, name systemic disadvantage, or use the language of transformation without redistributing power or disrupting organizational control. For example, practices that would reflect redistributing power, such as Aboriginal and Torres Strait Islander people holding executive and board level roles with genuine authority, were largely absent. This frame also reflected a pattern where companies appeared to take responsibility, but their actions were structured to protect their image, not to enact structural change. For instance, organizations used aspirational language, such as ‘reconciliation will be achieved through honest acknowledgment of and responsibility taken for the injustices of the past… and humility and compassion for addressing the challenges of the present’ (Org21), without specifying changes to corporate governance or decision-making power. Through symbolic gestures, externalized blame, and low-stakes reform, reconciliation became an act to perform, rather than a process of transformation. Within this frame are three subframes: ‘sanitized history, cultural endurance, and displaced responsibility’.

#### Sanitized history

The ‘sanitized history’ frame enabled organizations to divert attention away from their historical or ongoing involvement in health-harming commercial activities. This was particularly evident in sectors, such as mining, transport, and professional services, which have been linked to extractive activity, directly and through supply chains, harmful to Aboriginal and Torres Strait Islander communities, as well as in retail, food, and consumer industries that support extractive activities (Org1–5, 21–22, 23–33). Even when colonization and other past injustices were mentioned, they were framed as historical events and rarely connected to how the organization or industry continues to benefit from these systems (Org4, 17, 21, 24, 27, 35, 38). Additionally, the retail, food, and consumer industries, which engage in the supply chain of health-harming products, such as alcohol, tobacco and ultraprocessed foods, did not appear to reflect how their business practices contribute to Aboriginal and Torres Strait Islander health inequity (Lyons *et al*. 2021, Crocetti *et al*. 2023b, Leersen *et al*. 2024). Instead, feel-good phrases, such as ‘achieving the desired outcomes’ (Org38) ‘unified Australia’ (Org21), or ‘there is only one race, the human race’ (Org4), were deployed, omitting the ongoing structural inequities present for Aboriginal and Torres Strait Islander people and communicating a sanitized version of reconciliation.

#### Cultural endurance

The ‘cultural endurance’ frame was used by organizations to signal respect but also to position reconciliation as a response to the cultural longevity and survival rather than to historical and ongoing inequities. These references included descriptions, such as ‘strong’, ‘survived’ culture of ‘60 000 years’ (Org1, 3, 11, 26) ‘65 000 years’ (Org1, 38), and ‘100 000 years’ (Org40).

#### Displaced responsibility

A key feature of the ‘performative accountability’ frame was the displacement of responsibility, where organizations acknowledged barriers to reconciliation but positioned them as external to their own operations. This was evident in how some organizations framed economic inequity. Some RAPs acknowledged that Aboriginal and Torres Strait Islander businesses faced ‘structural, social, and economic barriers’ and lower average revenue than non-Indigenous (small to medium) businesses ($0.8 M vs $1.5 M), despite contributing $8.8 billion to the economy (Org35). Yet these disparities were framed as external problems, without questioning internal practices that sought to increase companies’ power and market share. The solution was performatively framed in terms of ‘helping’ Indigenous businesses, rather than addressing the corporate decision-making, market structures, or systemic racism that maintains exclusion.

Similarly, organizations operating in sectors associated with extractive activity, financial exploitation, or the supply of health-harming commodities seldom reflected on how their industry practices contribute to Aboriginal and Torres Strait Islander health inequities, instead framing reconciliation as a voluntary good-faith endeavour separate from core business operations. For example, one organization emphasized ‘voluntary social investment’ and ‘good faith negotiation’ in reaching Native Title agreements, reinforcing a narrative of ethical engagement without acknowledging the structural harms associated with the projects themselves (Org22).

Together, these examples illustrate how displaced responsibility operated as a framing device that acknowledged constraint without interrogating organizational agency. Reconciliation was positioned as vulnerable to external disruption or structural forces beyond corporate control, rather than as a commitment requiring internal reform, redistribution of power, or sustained institutional accountability.

### Symbolic leadership

Through the ‘symbolic leadership’ frame, organizations positioned themselves as leaders in reconciliation but rarely demonstrated it. Across RAPs, leadership discourse was invoked in different ways, including by organizations calling themselves leaders, working with external or community leaders, setting up pathways to leadership for internal staff, or using vague measures of leadership in progress reporting. This frame appeared more about looking credible and responsible than creating leadership and power in decision-making for Aboriginal and Torres Strait Islander people. This ‘symbolic leadership’ frame functioned to build credibility and moral capital, often reinforced through awards or certifications.

Rather than demonstrating substantive change, organizations positioned themselves as champions of reconciliation through repeated positive characterizations of themselves. This frame positioned companies as the leadership required ‘in responding to First Nations issues’ and a ‘true market leader in supplier diversity’. Within this frame were three subframes: ‘external or community-focused leadership, pathway leadership, and ambiguous leadership’.

#### External or community-focused leadership

Under ‘external or community-focused leadership’, leadership was defined through external relationships, partnerships, and influence rather than through internal authority or shared governance. Examples included ‘Engaging’ external Aboriginal and Torres Strait Islander leaders to guide development and implementation of our [RAP] (Org15), broader definitions such as ‘influential leadership’ that are not formal titles, but community or teams (Org8), and commitments to supporting emerging leadership in First Nations communities, rather than executive roles (Org7–8). This functioned to demonstrate connection and collaboration with Aboriginal and Torres Strait Islander peoples, positioning the organization as responsive to community voices while maintaining internal hierarchies.

#### Pathway leadership

Commonly promoted commitments to supporting Aboriginal and Torres Strait Islander leadership included partnerships, internships, and university scholarships (Org1, 34–35), signalling that an administrative internship may be a substantial step towards an executive position. Examples of Aboriginal and Torres Strait Islander people reaching executive or board level positions were extremely limited, with leadership representation largely confined to middle management roles.

#### Ambiguous leadership

Organizations used ‘ambiguous leadership’ to communicate and report on their own definitions of leadership, including conflating trade positions, such as tradespeople and technicians, with leadership positions under broad metrics functioning to exaggerate the visibility of Aboriginal and Torres Strait Islander leadership, e.g. stating they reached employment of ‘4102 Indigenous team members, including 223 Indigenous team members in trade or leadership roles’ (Org34). Another organization suggested apprenticeships as a pathway to leadership: ‘We have had several successes, such as apprentices moving into leadership roles to mentor new apprentices’ (Org1).

Several organizations claimed to be committed to Aboriginal and Torres Strait Islander leadership but did not set clear measurable targets. Of those that did set leadership targets, leadership was variously conceptualized, seldom as a senior executive within the company. For example, ‘By 2025 and in addition to our uplift of a minimum of 5% of our employees identifying as Aboriginal and/or Torres Strait Islander people we aim to see 5% of this cohort in leadership positions (band six and above, encompassing clinical roles and higher)’, ‘leaders can be people who have influence across community, teams and the company that may not fit our leadership definition, therefore we also aim to see 21% of people in positions of influential leadership’ (Org13) and ‘provide minimum of three opportunities for cultural learning delivered by Aboriginal and Torres Strait Islander organizations each year. This includes cultural immersion, bespoke lunchtime gatherings and/or leadership development training’ (Org4).

### Truth signalling

The ‘truth signalling’ frame was deployed in many RAPs to represent organizations as responsible and trustworthy. Words, such as ‘truth,’ ‘transparency,’ and ‘accountability’, were used repeatedly but often not followed through with tangible action. Many RAPs used language, such as ‘[organisation] has demonstrated maturity and transparency’, to suggest that openness and honesty were central to their reconciliation efforts. Others emphasized that ‘the report represents a step change in transparency for our business, which is critical for building trust’ (Org27) or that ‘we have to be transparent, and we have to have trust’ (Org22). This frequent use of transparency discourse provides a moral signal, framing the organization as trustworthy, reflective, and ethically sound. In most cases, these transparency claims only referred to internal processes or reports for select clients, not information openly shared with Aboriginal and Torres Strait Islander communities or the public. For example, one RAP mentioned ‘transparent data and analytics’, but these served mostly client interests, not broader transparency (Org31). This ‘transparency’ serves more as a framing function than inviting real scrutiny; what appears as integrity is closer to ‘truth signalling’.

Many RAPs also used truth-telling language to signal moral commitment and align with national reconciliation conversations, such as ‘speak into a national narrative of truth-telling and reconciliation’ (Org1) and ‘create a space for truth telling to build greater understanding and acceptance’ (Org33). While some organizations offered legitimate definitions of truth telling, the term was mostly focused on the context of education and training programmes. As a result, truth telling was often used to signal ethical responsibility and progressive values, rather than truth telling that is the practice of accurately and inclusively recounting Queensland’s history, recognizing the contributions of Aboriginal and Torres Strait Islander peoples and documenting their experiences of colonization, involving examining laws, policies, and practices and their impacts (State of Queensland 2025).

### Substantive recognition

Some RAPs reflected a deeper understanding of ongoing inequality, naming colonization, dispossession, intergenerational trauma, and institutional exclusion as drivers of harm. For example, one stated: ‘Archaeology has a historical legacy of grave robbing and relic theft and the historical traumas and present-day traumas (such as Juukan Gorge and many other key cultural sites being destroyed every single day in this country) present significant challenges for reconciliation’ (Org40). Another acknowledged the importance of ‘addressing the ongoing impacts of colonisation and systemic discrimination’ (Org23). These examples were rare, suggesting that ‘substantive recognition’ appeared only marginally within the dataset.

## Discussion

Our analysis revealed that organizations holding Stretch and Elevate RAPs in Queensland frequently frame reconciliation through ambitious claims and symbolic commitments while offering limited pathways towards transformative organizational change. These organizations were large and well resourced, operating in sectors with considerable influence over social, cultural, and CDoH, situating their RAPs as both influential and conflicted.

Across the frames identified in Table 2, organizations positioned themselves through moral leadership, corporate reputation, and managerial progress, aligning more closely with corporate interests and risk management than with Reconciliation Australia’s expectation to ‘uphold the self-determination of Aboriginal and Torres Strait Islander peoples and drive systemic and transformational change’ (Reconciliation Australia 2023). Executive-level Indigenous representation was minimal, and reporting transparency varied widely, shaping how reconciliation was framed and operationalized.

The ‘cultural endurance’ frame shifted attention away from the continuing harms and systemic injustices that require Indigenous people to be ‘strong’ and remain in ‘survival’ mode, while much of the Australian population does not face such burdens. Celebrating resilience in this way risks naturalizing these burdens and enabling non-Indigenous audiences to overlook inequities they do not experience.

RAP commitments reflected a distinctly commercial context shaped by neoliberalism, reframing social obligations as measurable managerial tasks. Employment targets, cultural awareness training, and supplier diversity dominated commitments because they produce quantifiable outcomes that align with existing corporate reporting frameworks. This logic emphasizes individual autonomy, market participation, and corporate efficiency while revealing little about the quality of outcomes, such as retention, leadership progression, or experiences of racism (Poirier *et al*. 2022). By defining reconciliation primarily through economic participation and managerial indicators, organizations risk reinforcing the same neoliberal systems that perpetuate Aboriginal and Torres Strait Islander inequity. Through a CDoH lens, ‘business-as-usual reconciliation’ facilitates continued supply of harmful commodities while sustaining policy environments that produce health inequities (Lencucha and Thow 2019).

A frequent discursive strategy was positioning organizations as the ‘leader’ or ‘champion’ of reconciliation. This placed them at the centre of the reconciliation narrative while masking the near absence of meaningful Aboriginal and Torres Strait Islander representation in executive and decision-making roles. ‘Symbolic leadership’ inflated progress by reporting trade or junior roles as leadership positions and presenting entry-level programmes as pathways to leadership, a pattern consistent with Weerasinghe *et al*. (2023). This framing relinquished responsibility to directly recruit into senior roles, ensuring leadership remained corporate and colonized. It speaks to a logic of ‘doing something’ rather than nothing.

A key finding of this study was that very few RAP organizations acknowledged the role their company or industry sector played in harming Aboriginal and Torres Strait Islander peoples, and when they did the emphasis was on the historical element. However, recent examples illustrate this contradiction. As a result of two reports from Australia’s financial regulatory authority, ‘Better banking for Indigenous consumers (Australian Securities and Investments Commission 2024b)’ and ‘Better and beyond: Expanding better banking outcomes to more low-income Australians (Australian Securities and Investments Commission 2025a)’, the Commonwealth Bank of Australia (CBA), which currently holds the highest level (Elevate) RAP and ‘continually seeks to be known as the culturally safe bank of choice’ [p. 9], was found to have charged approximately A$270 million in fees to around 2.2 million low-income customers, including at least A$25 million from First Nations people. Unlike the other three banks involved, CBA has not agreed to full refunds for all affected customers (Deves 2025, Financial Rights Legal Centre 2025).

Companies were more likely to ‘displace responsibility’ by downplaying the impact of their own industries, such as those involved in mining, banking, insurance, supermarkets, corrections, despite evidence that their products and practices contribute to poorer health outcomes for Aboriginal and Torres Strait Islander peoples. Rather than acknowledging these structural harms, their RAPs framed reconciliation as a voluntary, good-faith project rather than a non-negotiable moral and institutional obligation.

When organizations whose profit models are historically (and recently) tied to dispossession, environmental degradation, sales of harmful products, or exploitative labour practices adopt the language of reconciliation, they actively co-opt reconciliation as a mechanism of legitimacy rather than structural redress. In this sense, commercial actors use RAPs as instruments of reputation management to advance their power and profits. The substantial size and resources of these organizations further influence this dynamic, with most reporting annual revenues in the billions and employing workforces numbering in the thousands. The concentration of RAPs among these sectors illustrates who has the financial capacity and resources to participate at higher RAP levels, but also as the ‘leaders in reconciliation’, who defines the terms and boundaries of reconciliation itself. Our findings highlight a critical paradox: the RAP program’s success in engaging powerful corporate actors has elevated reconciliation within organizational settings while simultaneously enabling forms of corporate capture that narrow its scope and limit its capacity for structural change.

Another central finding was that organizational transparency within RAPs and associated reporting was limited, inconsistent, and often difficult to interpret. With organizations holding the power to self-report, they retain control over what is disclosed and how commitments are framed, enabling them to signal progress while maintaining authority over the narrative. Positive yet vague descriptors, such as ‘on track’ or ‘working towards’, create the appearance of accountability without requiring substantive evidence of change. This dynamic is reinforced by Reconciliation Australia’s reliance on anonymized, self-reported data in its annual Impact Report, which prevents organizational-level assessment and limits independent scrutiny. These patterns align with broader environmental, social, and governance (ESG) and CSR reporting literature, which consistently shows that organizations disclose extensively on socially desirable goals, yet the quality, comparability, and verifiability of data remain weak, producing symbolic rather than substantive change (Schepis 2020, Lodhia Kaur and Kuruppu 2023). In our findings, this gap between signal and substance meant that structural inequities remained largely unaddressed, while organizations demonstrated progress through symbolic gestures, managerial processes, and public reporting. This dynamic is central to our core finding that RAPs are often less about transforming systems and more about managing how reconciliation is seen, measured, and marketed (Schepis 2020, Lodhia Kaur and Kuruppu 2023, Bargallie Lentin and Turner 2025).

RAPs revealed how social causes can be absorbed into corporate strategy, functioning as an extension of broader CSR agendas. The current approach to RAPs allows organizations to manage moral credibility alongside profit, creating a narrative of ethical capitalism within a system of racial capitalism (Schepis 2020). Crocetti *et al*. demonstrated how organizations can allow, enable, or cause harm to Aboriginal and Torres Strait Islander peoples not only through the sale of harmful products, such as alcohol or tobacco, but also through practices, such as lobbying and marketing, including the use of RAPs as a strategic corporate tool (Crocetti *et al*. 2022, 2023a). Our study extends this evidence by uncovering how corporate activity that appears positive on the surface can coexist with, and even help to obscure, practices that cause or perpetuate harm.

Corporate framing within RAPs often took the form of ‘performative accountability’, where organizations signalled responsibility and awareness without enacting the structural changes that genuine accountability requires. Similar patterns appear in other colonial contexts, such as Canada, where reconciliation discourse is appropriated and commodified to reflect dominant institutional values rather than Indigenous views (Duhamel *et al*. 2024). This study used established framing, critical social theory, and CDoH approaches to analyse Stretch and Elevate RAPs, giving it broader relevance across sectors and jurisdictions. A key limitation of this study was its reliance on publicly available documents, which restricted our ability to assess how RAP commitments translate into lived experience and health outcomes.

The consequence is that reconciliation, originally intended to advance Aboriginal and Torres Strait Islander rights, has largely been reduced to a corporate product that can be bought, branded, certified, and publicly displayed. RAPs may do more to protect organizational legitimacy than to redistribute power or address structural harm, allowing organizations to define and control a process that affects Aboriginal and Torres Strait Islander health and wellbeing. These findings show that under corporate-led reconciliation, the purpose, definition, audience, and beneficiaries of RAPs are often shaped in favour of organizations rather than Aboriginal and Torres Strait Islander interests.

Viewed through the CDoH lens, RAPs are a corporate reputation management practice, part of the broader commercial playbook with potential health implications (Gilmore *et al*. 2023). They shape workplace inclusion, influence economic participation, and provide a platform for organizations to frame their practices, whether harmful or beneficial to health. While this study highlights the limits of voluntary corporate self-reporting, mandatory ESG disclosure may offer stronger corporate accountability. In Australia, ESG disclosure for large ASX-listed companies (listed on the Australian Securities Exchange) is transitioning to a new framework that mandates annual self-reporting on climate-related financial information for financial years starting in 2025 and will expand to smaller companies over the next 3 years (Australian Securities and Investments Commission 2025b). As such, the first round of reports has not yet been completed, but Australian Securities and Investments Commission has indicated that, in future, this mandatory reporting may extend to other sustainability-related disclosures (Australian Securities and Investments Commission 2024a).

If Reconciliation Australia were to adopt a similar mandatory reporting regime, the implications would be considerable. Standardized and verifiable reporting could strengthen public accountability, reduce performative transparency, and allow meaningful comparison of organizational progress across sectors. Greater scrutiny would likely narrow participation; however, this reduction may be productive rather than problematic, distinguishing organizations prepared to subject themselves to formal accountability from those engaging in reconciliation primarily as reputational risk management.

A mandatory framework could also risk transforming reconciliation into another compliance exercise, where the focus shifts from systemic change and community priorities to corporate performance indicators. Independent impact reporting, conducted separately from organizational self-reporting, offers one way to strengthen accountability without defaulting to compliance logic. The challenge, therefore, would be to design any mandatory reconciliation reporting framework that enhances accountability without erasing the relational and justice-based principles that reconciliation is meant to uphold.

From a commercial determinant of health perspective, this study demonstrates that RAPs function as reputational shields, permitting organizations to claim their investments in reconciliation while structural relations of power and profit remain intact. The frames identified reproduce core dynamics described across the CSR and CDoH literature and extend these specifically to the Aboriginal and Torres Strait Islander context (Schepis 2020, Gilmore *et al*. 2023, Bargallie Lentin and Turner 2025).

## Conclusion

This study demonstrates that corporate reconciliation is often framed in ways that reinforce existing power imbalances. RAPs are positioned as tools for reputation management, accountability signalling, and corporate strategy, allowing organizations to demonstrate commitment while maintaining power. Few RAPs acknowledged the role of these organizations in structural inequities.

While RAPs provide visibility and entry points for dialogue, they also enable corporations to control the reconciliation narrative. Strengthening accountability requires transparent, comparable, and independently verified reporting, alongside Aboriginal and Torres Strait Islander-led governance. Without that leadership, mandatory disclosure risks becoming another compliance exercise.

Overall, RAPs are less about transforming systems and more about managing how reconciliation is seen, measured, and marketed. Structural inequities remain unchallenged, while progress is often framed through symbolic gestures, managerial processes, and selective reporting. The question is now whether corporate reconciliation can move beyond symbolic performance towards genuine structural reform.

## Supplementary Material

daag061_Supplementary_Data

## Data Availability

This analysis is based on data from publicly available websites.
