# Supplementary material for: Reconciliation or reputation: critical analysis of commercial sector commitments and framing in reconciliation action plans
Source: Health Promot Int. 2026 May 8;41(3):daag061. doi: 10.1093/heapro/daag061 (PMC13155105; doi:10.1093/heapro/daag061)
Supplement: daag061_Supplementary_Data [file daag061_supplementary_data.docx]

**Organisation profiles**

| *RAP Organisation Data Sources* | Portfolio | | Resources | | Organisation | | | Transparency | | |
| --- | --- | --- | --- | --- | --- | --- | --- | --- | --- | --- |
|  | **Industry** | **Products and Services** | **Employees** | **Annual Revenue** | **Organisation Structure** | **Aboriginal and Torres Strait Islander representation (Board/Executive)** | **RAP Governance (1=Leadership/Decision-making)** | **Mention of harm to Aboriginal and/or Torres Strait Islander people within the RAP** | **RAP reporting method** | **Stated commitment to monitoring and accountability** |
| *Org1 Stretch RAP (2023-2026)*  *Annual Report (2024)* | Construction, infrastructure and manufacturing | Defence and social infrastructure, infrastructure, telecommunications, transport | 35,000 (AUS and NZ) | A$6.1b | ASX Listed Australian | Not listed/unclear | Lead/Decision-making unclear  RAP Working Group  Indigenous Advisory Board  RAP Champion (Group Executive – People, Safety and Culture) | “we will commit to actions that support a future where the wrongs of the past are both understood and never repeated and where truth, justice, healing and historical acceptance are strengthened”  “address the barrier of minor criminal records to Aboriginal and Torres Strait Islander employment”  “27 candidates were employed within [Org1] that were initially rejected based on their criminal history” | Integrated  Annual Report | No clear accountability measures  “[Org] holds [Org1] accountable to the actions of the RAP” |
| *Org2 Stretch RAP (2023-2026)*  *Sustainability Report (2024)* | Construction, infrastructure and manufacturing | Engineering, construction, and asset management services across various sectors including infrastructure, building, and rail | 3,000+ | £4.8bn | Private Multinational | Not listed/unclear | Lead/Decision-making unclear  RAP Working Group | No clear mention of harm  “rich cultural history” “we have a long history of supporting Indigenous businesses...” “Showcasing history of the local area” “engage in histories by celebrating NAIDOC” | Integrated  Sustainability Report  Highlights they are making “efforts” nothing measurable | No clear accountability measures |
| *Org3 Stretch RAP (2022-2025)*  *Annual Review (2024)* | Construction, infrastructure and manufacturing | Engineering-led construction services across various sectors, including infrastructure, building, rail, resources, and energy. | 39,000 | A$16.8b | Public Multinational | Not listed/unclear | Lead/Decision-making unclear  RAP Working Group | No clear mention of harm | Integrated  Annual Review | No clear accountability measures |
| *Org4 Stretch RAP (2022-2025)*  *Annual Report (2024)* | Construction, infrastructure and manufacturing | Engineering, construction, maintenance, and industrial services to the resources, energy, and infrastructure sectors | 7,500 | A$1.9billion | ASX Listed Multinational | Not listed/unclear | Lead/Decision-making conflicted  RAP Committee overseen by Managing Director and Diversity and inclusion Champion.  “RAP Committee oversee the direction of our journey toward reconciliation and meet quarterly to monitor and report against our progress” | No clear mention of harm  Acknowledges challenges in accessing work-ready Aboriginal and Torres Strait Islander employees | Integrated  Annual Report | No clear accountability measures |
| *Org5 Stretch RAP (2022-2025)*  *Climate and Sustainability Solutions (2024)* | Construction, infrastructure and manufacturing | Engineering, construction, operations, and maintenance services to the energy, transportation, resources, communications, and defence sectors | 9,000 | Not listed/unclear | Private Multinational | Not listed/unclear | Lead/Decision-making unclear  RAP Committee | No clear mention of harm  References 'appreciating' Aboriginal and Torres Strait Islander cultures | Integrated  Climate and Sustainability Solutions | No clear accountability measures |
| *Org6 Stretch RAP (2023-2026)*  *Sustainability Report (2024)* | Construction, infrastructure and manufacturing | Operates and manages the high voltage electricity transmission network in NSW and the ACT, connecting generators, distributors, and major end users | Not listed/unclear | Not listed/unclear | ASX Listed Australian | Not listed/unclear | Lead/Decision-making unclear  RWG and RAP Program Team | No clear mention of harm | Integrated  Sustainability Report | No clear accountability measures |
| *Org7 Stretch RAP (2022-2025)*  *RAP Progress Report (2024)*  *RA Statement (2021)*  *Annual Report (2024)* | Construction, infrastructure and manufacturing | Telecommunications including mobile, internet, and entertainment | Not listed/unclear | A$23.5b | ASX Listed Australian | Not listed/unclear | Lead/Decision-making unclear  RAP Working Group | No clear mention of harm (RAP)  “At no time did [Org7] alert us [RA] to the complaints covered by the ACCC investigation” “and did not notify RA in advance of the public announcement of the proposed settlement” (RA) | RAP Progress Report | No clear accountability measures  Reports internal mechanisms for monitoring RAP |
| *Org8 Elevate RAP (2020-2024)*  *Annual Report (2024)* | Construction, infrastructure and manufacturing | Construction, infrastructure, and real estate services | 10,000 | A$9.3b | Public Multinational | Not listed/unclear | 1. RAP Expert Panel, Reconciliation Australia & [Org8] Board  2. All Australian Lendlease employees, RAP Passionates, RAP Steer Co & RAP Working Group, [Org8] Business Leaders | “by focussing on telling the truth of place, the industry can drive an understanding and acceptance of past wrongs and the impacts on First Nations peoples.” | Integrated  Annual RAP report (1 page, includes 3 goals). In contrast, the RAP is 57 pages | No clear accountability measures |
| *Org9 Stretch RAP (2024-2027)*  *Sustainability Report (2024)* | Financial and insurance | Investment management, asset management, and financial services | Not listed/unclear | Not listed/unclear | Private Australian | Not listed/unclear | Lead/Decision-making unclear  RAP Working Group | “economic participation is a critical factor contributing towards equal lifestyle, health and educational opportunities for all Australians and that through historical government policy and practice, our First Nations peoples have largely been denied that opportunity” | Integrated  Sustainability Report | No clear accountability measures |
| *Org10 Stretch RAP (2024-2026)*  *Annual Report 2024* | Financial and insurance | Roadside assistance, insurance, car servicing, motoring services | 5,000 | A$963.742m | Private Australian | Not listed/unclear | 1. Board of Directors  2. GCNC approval  3. First Nations Advisory Group  4. CEO/Executive Leadership Team  5. RAP Steering Committee | No clear mention of harm  “appreciation for the histories and rich cultures”  “we’re still young in the grand scheme of our country’s history” | Internal only | No clear accountability measures |
| *Org11 Stretch RAP (2023-2026)*  *Sustainability and ESG Governance Annual Report (2023)*  *Annual Report (2024)* | Financial and Insurance | Health insurance, aged care, dental care, optical care, and other health services | 15,000 (Australia, excluding contractors) | £16.9b | Private Multinational | Not listed/unclear | Lead/Decision-making unclear  RAP Working Group | Vague  “reflect on the true histories and challenges faced by Aboriginal and Torres Strait  Islander peoples, families and communities” | Integrated  ESG Reporting | No clear accountability measures  “commitment to accountability” |
| *Org12 Services Ltd Stretch RAP (2023-2026)*    *Sustainability Report (2024)* | Financial and Insurance | Banking  Superannuation  Retirement planning  Wealth management  Financial advisory | 6,000 | A$3.2b | AXS Listed  Australian | Not listed/unclear | 1. Exec Committee & Board  2. Inclusion and Diversity Council & Superannuation Trustee Board  3. RAP Committee & Indigenous Employee Network  4. RAP Champions & RAP Working Group | No direct mention of historical harms or remediation efforts | Integrated  Sustainability Report | No clear accountability measures |
| *Org13 Stretch RAP (2022-2025)*  *Impact Report (2024)* | Financial and Insurance | Financial Healthcare  Community services | 7,000 | A$2b | Private Australian | Not listed/unclear | Lead/Decision-making unclear  RAP Leadership Team, Reconciliation Working Group, co-design with Aboriginal and Torres Strait Islander people, internal and external stakeholders, Secretariat, General Manager, Indigenous Business & Community Engagement, CEO, Secretariat, Head of Internal Communications, Head of Brand and Content, Head of Digital Channels, RAP Leadership Team, Group Managing Director, Head of Organisational Development, Head of Digital Channels, Head of Learning | “However, we recognise there is much more to do—as a  company and as a country—if we are to truly alleviate  and amend the pain and suffering inflicted on Aboriginal  and Torres Strait Islander peoples over the two centuries  that followed European colonisation.”  “involves shared  knowledge and understanding of the history, experience  and ongoing challenges faced by Aboriginal and Torres  Strait Islander peoples.” | Integrated  Impact Report | No clear accountability measures  “Our governance approach also  includes the monitoring and ongoing evaluation of our actions” |
| *Org14 Stretch RAP (2022-2025)*  *Annual Report (2024)* | Financial and Insurance | General insurance services | 13,500 | A$12b+ | Public Australasian | Not listed/unclear | Lead/Decision-making unclear  RAP Working Group, RAP Steering Committee | No clear mention of harm | Integrated  Annual Report  “Continued progress in delivering our Stretch Reconciliation Action Plan 2022-2025 across its four areas”  Has 1 measurable commitment: “sponsored a senior employee to attend the Executive Indigenous Leaders program run by UNSW Business School” | No clear accountability measures |
| *Org15 Elevate RAP (2023-2025)*  *RAP Progress Report (2024)*  *Annual Report (2024)* | Financial and Insurance | Comprehensive banking and financial services | 48,000 | A$9,481m | Public Australian | Not listed/unclear | Lead/Decision-making unclear  Indigenous Advisory Council | “Historical Acceptance: Understanding the role the financial  services sector played in Indigenous economic disadvantage so  we are able to acknowledge the past and move forward towards  reconciliation as a unified nation.” | RAP Progress Report | No clear accountability measures |
| *Org16 Elevate RAP (2022-2025)*  *Annual Report (2024)* | Financial and insurance | Comprehensive banking and financial services | 35,240 | Not listed/unclear | ASX Listed Australian | Not listed/unclear | Lead/Decision-making unclear  RAP Steering Committee; Indigenous Advisory Group | Not listed/unclear | Integrated  Annual Report  States “publicly report against our RAP commitments annually, outlining achievements, challenges, and learnings” - Doesn't say where this information is publicly available | No clear accountability measures |
| *Org17 Stretch RAP (2023-2026)*  *Sustainability and ESG Governance Annual Report (2023* | Legal Services | Legal services, pro bono work, community engagement | 1,489 (FTE) | Not listed/unclear | Private Australian | Not listed/unclear | Lead/Decision-making unclear  RAP Working Group, consultation with partners and employees, RAP Office Committees, First Nations employees, internal practice groups, departments involved in RAP initiatives | No clear mention of harm | Integrated  ESG report includes some RAP commitments, but no detailed metrics or progress | No clear accountability measures  “While we recognise our achievements we also acknowledge there have been challenges” |
| *Org18 Stretch RAP (2024-2027)*  *Societal Impact Deep Dive (2024)*  *Global Financial Results (2024)* | Legal Services | Legal services, pro bono work, corporate advisory, Indigenous advocacy programs | Not listed/unclear | US$4.2b | Private Multinational | Not listed/unclear | Lead/Decision-making unclear  RAP Working Group | “our role is to nurture trust and support  Aboriginal and Torres Strait Islander-led solutions to undo and address the  wrongs caused by racism and colonisation” | Integrated and limited, however, website features Indigenous advocacy and pro bono work - integrated into broader ESG commitments not just as part of the RAP | No clear accountability measures |
| *Org19 Stretch RAP (2023-2025)*    *Responsible Business Report (2024)*    *Annual Business Report (2024)* | Legal Services | Law firm including commercial and corporate  Litigation services | 1,600 (Australia) | £961m | Private  Multinational | Not listed/unclear | Lead/Decision-making unclear  Board and/or Executive team member, RAP Leadership Circle, First Nations Lead, Global Pro Bono and Societal Impact Partner, People & Culture Director, Office of General Counsel, Spectrum Network | Acknowledges historical disadvantages and systemic barriers | Integrated  Responsible Business Report  Some RAP commitments “recording over 65,000 hours of pro bono work on 683 pro bono matters with 1,300 partners and staff volunteering their time” | No clear accountability measures |
| *Org20 Stretch RAP (2022-2025)*  *Annual Litigation Trends Survey (2024)* | Legal Services | Comprehensive legal services, dispute resolution, regulatory, and international advisory services | Not listed/unclear | Not listed/unclear | Private Multinational | Not listed/unclear | Lead/Decision-making unclear  RAP Working Group | “hear and become more aware of Australia’s First Nations peoples and culture, the impact of  colonisation, and the proud and ancient culture that still remains.” | Integrated  Annual Survey | No clear accountability measures |
| *Org21 Stretch RAP (2023-2026)*  *RAP Progress Report (2024)*  *BP Annual Report (2024)* | Mining | Energy products and services, including fuel, lubricants, and renewable energy solutions. | 100,500 | A$27.3b | Private Multinational | Not listed/unclear | Lead/Decision-making unclear  RAP Working Group, Indigenous Business Resource Group | “We believe reconciliation will be achieved through honest acknowledgment of and responsibility taken for the injustices of the past, humility and compassion for addressing the challenges of the present and First Nations’ sovereignty and unity as we look towards the future” | RAP Progress Report | No clear accountability measures |
| *Org22 Elevate RAP (2023-2027)*  *Australian Indigenous Social Investment Report (2024* | Mining | Resources sector, focusing on mining and extraction of minerals including coal, iron ore, and copper | 91,587  (49,892 Australia) | US$55,658m | ASX Listed Multinational | Not listed/unclear | Lead/Decision-making unclear  Board Sustainability Committee, Executive Leadership Team, Minerals Australia Leadership Team, Australian Indigenous Peoples Working Group, Asset Leadership Teams, Traditional Owner Agreement and Heritage Committees | “all Australians understand and accept the wrongs of the past and the impact of these wrongs. Australia makes amends for the wrongs of the past and ensures these wrongs are never repeated”  “implementing a more structured mechanism to better understand [Org22]’s historical relationships with Aboriginal and Torres Strait Islander peoples and inform exploration of truth-telling processes” | Integrated  Social Investment Report | No clear accountability measures  “incorporate Aboriginal and Torres Strait Islander people’s perspectives, values and knowledge in each applicable stage of evaluation to support improvements and provide accountability” |
| *Org23 Stretch RAP (2024-2027)*  *Annual Reports and Accounts (2024)* | Professional services | government services across sectors such as justice, immigration, defence, transport, and health | 8,000 | >A$1B | ASX Listed Multinational | Not listed/unclear | Lead/Decision-making unclear  RAP Working Group. RAP Chair is Ngarigo/Yuin | “ongoing impacts of colonisation, including how this relates to the many industries that [Org23] works within, which have distinct colonial histories which have ongoing effects to this day” | Integrated  Annual Report  Has one paragraph stating that they launched their fourth RAP. And are developing a RAP for [Org23] Canada. Limited metrics | No clear accountability measures |
| *Org24 Stretch RAP (2024-2027)*  *Reconciliation at Nous (ND)*  *Our People (ND)* | Professional services | Consulting services across various sectors (education, government, health) | 700 | Not listed/unclear | Private Australian | Not listed/unclear | Lead/Decision-making unclear  RAP Working Group | “that colonisation of  Australia has resulted in disruption and trauma for  Aboriginal and Torres Strait Islander peoples.” | Integrated  No metrics | No clear accountability measures |
| *Org25 Stretch RAP (2023-2026)*  *Indigenous Engagement (ND)*  *ESG Report (2023)* | Professional Services | Integrated facility management services (Real estate, energy, technical, project delivery) | 2,500 | CA$83.7m | Private Multinational | Not listed/unclear | Lead/Decision-making unclear  National Indigenous Engagement Manager,  RWG, President & Managing Director/Exec Director, First Nations Employee advisory committee | “Strong focus on historical acceptance”  “Reconciliation means informing and empowering all people to participate in and contribute to reconciliation and addressing inequity and the acknowledgement of historical crimes committed. [Org25] employee, national survey, 2022” | Integrated  ESG Reporting  Other reporting included metrics similar to RAP commitments for Canada and North America but not for Australia | No clear accountability measures |
| *Org26 Stretch RAP (2022-2025)*  *First Nations peoples embracing apprenticeships report (2024)*  *First Nations people (ND)* | Professional Services | offers apprenticeship and traineeship services, recruitment solutions, and employment services across Australia | Not listed/unclear | Not listed/unclear | Private Australian | Not listed/unclear | Lead/Decision-making unclear  RAP Working Group:  - Aboriginal and Torres Strait Islander Advisor  - Co-chaired by National Indigenous Programs Manager and Aboriginal and Torres Strait Islander lead  - Four staff positions  - Four identified positions | “acknowledging  Aboriginal and Torres Strait Islander peoples as the First Peoples of this  land, and recognising that their ancestors were dispossessed, persecuted  and oppressed as a result of colonisation.”  “Achieving reconciliation involves acknowledging and addressing the  historical and often intergenerational injustices and inequities experienced  by Aboriginal and Torres Strait Islander peoples since colonisation. It is  equally important to acknowledge the strength and resilience shown” | Integrated  Multiple sources | No clear accountability measures |
| *Org27 Elevate RAP (2021-2025)*  *RAP Progress Report (2023)*  *Annual Report (2024)* | Professional Services | Audit, tax, consulting, advisory services | 10,000 | US$38b | Private Multinational | Not listed/unclear | Lead/Decision-making unclear  RAP Steering Committee | No clear mention of harm | RAP Progress Report  Provided a submission to the NIAA Indigenous Procurement Policy reform discussion paper | No clear accountability measures |
| *Org28 Stretch RAP (2024-2027)*  *First Nations Engagement (ND)*  *Annual Report (2024)* | Property Development/ Management | Integrated facilities services including cleaning, maintenance, catering, security, and support services. | 125,208 (“Asia & Pacific”) | A$5,279m | Private Multinational | Not listed/unclear | Lead/Decision-making unclear  Indigenous Engagement Manager, RWG, RAP Committee | “Placemakers will touch on and identify how the  impacts of intergenerational trauma effects things such as leave provisions  s (cultural leave, Sorry Business),” | Integrated  Annual Report  “Every year we report on the progress we have made as good corporate citizens,”  Has some case studies on webpage. | No clear accountability measures |
| *Org29 Stretch RAP (2023-2026)*  *Annual Report (2024)* | Property development/management | Residential, commercial and retirement property development and management | 1,100 | A$3,313m | ASX Listed Australian | Not listed/unclear | 1. Board of Directors (Oversight)  2. Group Sustainability Steering Committee (Leadership and strategic governance)  3. Reconciliation Leadership Team (Leadership, decision-making and endorsement)  4. Reconciliation Working Group (Delivery and implementation)  5. Reconciliation Ally Network (Advocacy and support) | No clear mention of harm | Integrated  Annual Report | No clear accountability measures |
| *Org30 Stretch RAP (2023-2026)*  *Annual Report (2024)* | Property development/management | Development, sustainability, financial, property, retail | Not listed/unclear | A$988.7m | ASX Listed Australian | Not listed/unclear | Lead/Decision-making unclear  RAP Working Group | No clear mention of harm | Integrated  Annual Report | No clear accountability measures |
| *Org31 Stretch RAP (2022-2025)*  *Social Agenda Report (2024* | Retail, food, and consumer | Professional services, workplace and office products, print and marketing services | Not listed/unclear | Not listed/unclear | ASX Listed Australian | Not listed/unclear | Lead/Decision-making unclear  RAP Working Group | Not listed/unclear | Integrated  Social Agenda Report | No clear accountability measures |
| *Org32 Stretch RAP (2022-2025)*  *ESG Report (2024)*  *Annual Results (2024)* | Retail, food, and consumer | Energy services, engineering, technology, energy | 220,000 (5,750 AUS and NZ) | €44,692m | Private Multinational | Not listed/unclear | 1. CEO and MD  2. RAP Executive Sponsor  3. National RAP Working Group  4. Waste RAP Working Group, Water RAP Working Group, Industrial and Energy RAP Working Group | No clear mention of harm | Integrated  ESG Report | No clear accountability measures |
| *Org33 Stretch RAP (2022-2025)*  *Sustainability Report (2024)*  *Annual Report (2024)* | Retail, food, and consumer | Food, supply chain and support services | 7,155 permanent | A$42.4b | Private Multinational | Not listed/unclear | Managing Director, Australian Leadership Team, Diversity and Inclusion Advisory Committee, First Nations Working Group, National General Manager, Diversity and Inclusion Unit, Managers and Supervisors, including Executive Directors and General Managers | No clear mention of harm | Integrated  Sustainability Report | No clear accountability measures |
| *Org34 Stretch RAP (2024-2027)*  *Sustainability Report (2024)* | Retail, food, and consumer | “[Org34] is one of Australia’s leading retailers,  with an extensive national supermarket and  liquor store footprint”  “physical outlets comprising  almost 850 supermarkets, over 950 Liquorland,  First Choice Liquor Market and Vintage Cellars stores,  and our online services.” | 120,000 (Australia) | A$43.6b | Public Australian | Not listed/unclear | 1. Board  2. Executive Leadership Team  3. Sustainability Steering Committee, RAP Steering Committee  4. RAP Working Group | No clear mention of harm  “From supporting communities in need, to supporting Aboriginal and Torres Strait  Islander organisations to grow and succeed, [Org34] has a proud history in this space and one which we are  pleased to continue.” | Integrated  Sustainability Report has some items related to RAP commitments | No clear accountability measures |
| *Org35 Elevate RAP (2022-2025)*  *Advancing Reconciliation (2024)*  *Annual Report (2024)* | Retail, food, and consumer | Raw materials, rent, freight, retail, financial, professional services. | Not listed/unclear | A$44.2b | ASX Listed Australian | Not listed/unclear | 1. Board  2. Managing Director and Executive General Manager, Corporate Affairs  3. Reconciliation Action Plan Steering Committee  4. Indigenous Network, Senior Management | “Aboriginal and Torres Strait Islander businesses have emerged as powerful  platforms towards self-determination for Aboriginal and Torres Strait Islander peoples, largely because one  of the many things that have survived colonisation are Aboriginal and Torres Strait Islander cultural values” | Integrated  Multiple sources | No clear accountability measures |
| *Org36 Stretch RAP (2023-2025)*  *Midpoint Progress Report* | Science, technology and engineering | Technology, science, engineering | Not listed/unclear | Not listed/unclear | Private Multinational | Not listed/unclear | Lead/Decision-making unclear  RAP Working Group including senior and local and overseas internal stakeholders, volunteer base, Executive Sponsor Committee, “External Advisory Board of trusted Indigenous Advisors” | No clear mention of harm | RAP Midpoint Report | No clear accountability measures |
| *Org37 Stretch RAP (2024-2027)*  *Community Report (2024)*  *Annual Report (2024)* | Sport | NRL Club | Not listed/unclear | A$75.2m | Private Australian | Not listed/unclear | Lead/Decision-making unclear  Board, RAP Champion, RAP Working Group, First Nations’ football family, external stakeholders, Reconciliation Australia | “taking into honest account, the root causes  that underpin intergenerational trauma that continues to be  manifested as result of colonisation”  “ensure our club remains free from  racism and all forms of discrimination, harassment and  violence. If something isn’t working, we will change it” | Integrated  Community Report | No strategies noted for RAP underperformance |
| *Org38 Elevate RAP (2024-2028)*  *Annual Report (2024)* | Sport | Sporting community, entertainment, hospitality | Not listed/unclear | A$43.7m | Private Australian | Not listed/unclear | Lead/Decision-making unclear  RAP Working Group, Indigenous Players Advisory Group, Women Indigenous Players Advisory Group | “Historian Project published and amplify research findings via  [Org38] owned channels, communicating the true history in the context of Rugby League.” | Integrated  Annual Report | No clear accountability measures |
| *Org39 Stretch RAP (2022-2025)*    *Sustainability Report (2024)* | Transport | Road and rail transport including of mining, primary producers, international and domestic markets | 5,930 | A$3,844m | ASX Listed Australian | Not listed/unclear | 1. MD & CEO, Executive Leadership Team  2. Indigenous Reference Group (IRG), People Strategy & Planning Team  3. Frontline leadership, employees, RAP champions | No clear mention of harm  “[Org39] has a proud history of  working in partnership with and  growing opportunities for First  Nations peoples” | Integrated  Sustainability Report | No clear accountability measures |
| *Org40 Stretch RAP (2024-2027)* | Professional services | Archaeology and cultural heritage | 10 | Not listed/unclear | Private Australian | Yes | Lead/Decision-making unclear | Not listed/unclear | Not listed/unclear | No clear accountability measures |
